# Peritonitis

**Published:** 1879-10

**Authors:** 


PERITONITIS.—Some one writing from Warrenville under date of August 28th, but who forgets to sign his name to his letter, gives the following outline of a case of peritonitis. In regard to the question of perforation of the intestines, suggested by the writer, we would suggest that the symptoms given are much more indicative of *suppuration* throughout the peritoneal surface, than of intestinal perforation.

Those who have seen many cases of acute peritonitis know that at the end of three or four days the fever and pain abate, presenting a deceptive appearance of improvement, to be speedily followed by either complete collapse, or suppuration, with cold extremities, feeble pulse, and daily partial febrile reactions, ending in fatal exhaustion in three or four days. If perforation of the intestine had taken place on the morning of the fifth day, as suggested by the writer, there should have been a sudden and severe increase of pain, followed by vomiting, collapse, and death, generally within 24 to 48 hours. The case given is as follows:

"Mrs. Jane M. Curtiss, of this village, died last Tuesday p.m. after a brief and painful sickness of one week. Her disease was acute peritonitis. The entire peritoneal membrane seemed to be involved. She had sickness and vomiting, and at first, suppression of urine, and later retention. Besides the usual pain and distension of the abdomen, there was an uncommon degree of tenderness and sensitiveness over the whole surface even to the hands and feet. All these symptoms gradually subsided by the fourth day. On the morning of the fifth day she was quite comfortable. About nine o'clock she had a light bilious evacuation, not unhealthy in appearance. She immediately grew worse, began to sink, her hands and feet were cold and clammy, and she did not rally under the stimulants and quinine which she took freely. She had a slight paroxysm of fever about noon every day, and sunk lower after it every day till she died, on the evening of the eighth day. Her most severe pain from the beginning was in the region of the right iliac fossa.

Is it not probable that there was a perforation of the intestine at that point, and that it took place when the movement of the bowels occurred on the morning of the fifth day of her sickness? A post mortem examination was out of the question, as she was a large and corpulent subject, and they wished to carry the remains to Peoria for interment. There was evidently a malarial influence operating through her whole sickness. I would like to have you insert this notice or the the substance of it in the JOURNAL. It may elicit an answer from some one that has had a similar case, confirmed by a post mortem examination."

